# Assessing the ability of an instrumental variable causal forest algorithm to personalize treatment evidence using observational data: the case of early surgery for shoulder fracture

**DOI:** 10.1186/s12874-022-01663-0

**Published:** 2022-07-11

**Authors:** John M. Brooks, Cole G. Chapman, Sarah B. Floyd, Brian K. Chen, Charles A. Thigpen, Michael Kissenberth

**Affiliations:** 1 Center for Effectiveness Research in Orthopaedics - Arnold School of Public Health Greenville, 915 Greene Street #302D, 29208, Columbia, SC 29208-0001 USA; 2 Health Services Policy & Management, University of South Carolina Arnold School of Public Health, Columbia, USA; 3 Department of Pharmacy Practice and Science, University of Iowa, Iowa City, USA; 4 Center for Effectiveness Research in Orthopaedics, Greenville, USA; 5 Clemson University College of Behavioral Social and Health Sciences, Public Health Sciences, Clemson, USA; 6 ATI Physical Therapy, Greenville, USA; 7 Prisma Health, Steadman Hawkins Clinic of the Carolinas, Greenville, USA

**Keywords:** Instrumental Variable Causal Forest Algorithm, Classification and regression trees (CART), Two-stage least squares (2SLS) estimators, Proximal humerus fracture, Surgery

## Abstract

**Background:**

Comparative effectiveness research (CER) using observational databases has been suggested to obtain personalized evidence of treatment effectiveness. Inferential difficulties remain using traditional CER approaches especially related to designating patients to reference classes a priori. A novel Instrumental Variable Causal Forest Algorithm (IV-CFA) has the potential to provide personalized evidence using observational data without designating reference classes a priori, but the consistency of the evidence when varying key algorithm parameters remains unclear. We investigated the consistency of IV-CFA estimates through application to a database of Medicare beneficiaries with proximal humerus fractures (PHFs) that previously revealed heterogeneity in the effects of early surgery using instrumental variable estimators.

**Methods:**

IV-CFA was used to estimate patient-specific early surgery effects on both beneficial and detrimental outcomes using different combinations of algorithm parameters and estimate variation was assessed for a population of 72,751 fee-for-service Medicare beneficiaries with PHFs in 2011. Classification and regression trees (CART) were applied to these estimates to create ex-post reference classes and the consistency of these classes were assessed. Two-stage least squares (2SLS) estimators were applied to representative ex-post reference classes to scrutinize the estimates relative to known 2SLS properties.

**Results:**

IV-CFA uncovered substantial early surgery effect heterogeneity across PHF patients, but estimates for individual patients varied with algorithm parameters. CART applied to these estimates revealed ex-post reference classes consistent across algorithm parameters. 2SLS estimates showed that ex-post reference classes containing older, frailer patients with more comorbidities, and lower utilizers of healthcare were less likely to benefit and more likely to have detriments from higher rates of early surgery.

**Conclusions:**

IV-CFA provides an illuminating method to uncover ex-post reference classes of patients based on treatment effects using observational data with a strong instrumental variable. Interpretation of treatment effect estimates within each ex-post reference class using traditional CER methods remains conditional on the extent of measured information in the data.

**Supplementary Information:**

The online version contains supplementary material available at 10.1186/s12874-022-01663-0.

## Introduction

Policy makers want patients to have *personalized evidence *when making treatment decisions [1–3]. The need for personalized evidence follows from recognition that *treatment effect heterogeneity* across patients is the rule rather than exception in healthcare [4–10]. While randomized controlled trials (RCTs) are the gold standard for evidence generation, with treatment effect heterogeneity it is difficult for RCTs to generate personalized evidence for many patients [4, 11–14]. Comparative effectiveness research (CER) using large observational databases has been suggested as an alternative to develop personalized evidence [1, 2, 15–18]. Observational data provide the perspective of real-world practice-based evidence and a diversity of patients well beyond those evaluated in RCTs [2, 3, 11, 17, 18]. However, inferential difficulties exist using traditional CER estimation approaches to provide personalized evidence. With traditional CER approaches the evidence for an individual patient is generally an average treatment effect estimate for a population subgroup or a “reference class” based on a combination of measured baseline factors *specified prior to estimation *[19–21]. Specifying appropriate reference classes a priori has been described as “the central problem when using group evidence to forecast outcomes (or treatment effects) in individuals.”[20] Even with a small number of measured baseline factors, a patient could be placed in an “indefinite number of subgroups” [19–21], and is often unclear which reference class is best aligned to each patient [4, 10, 19, 20, 22–29] Risk of misalignment is the greatest when using “one-variable-at-time” subgroups (e.g. young vs. old), as important distinctions across patients within a subgroup can be blended and lost [19–21]. In addition, because treatment variation in observational data reflects *choices* instead of random assignment, unmeasured factors affecting both treatment choice and outcomes can confound estimation and lead to biased treatment effect estimates within each reference class [30, 31].

A novel Instrumental Variable Causal Forest Algorithm (IV-CFA) described by Athey and colleagues has been developed with the potential to alleviate these issues [32]. Other similar instrumental variable-based causal forest algorithms have been developed [33, 34]. Causal forest algorithms (CFA) evolved from standard classification and regression tree (CART) and random forest ensemble methods with an objective to estimate personalized treatment effects without the need to specify reference classes a priori [32, 35–38]. However, CFA estimators require researchers to specify algorithm parameters related to tree construction and forest sizes prior to estimation. In addition, when applied to observational data, CFAs suffer the same bias risk from unmeasured confounding that threatens traditional CER estimators. To reduce confounding risk when using observational data, IV-CFA estimates individual treatment effects *using only the treatment variation in a population associated with an instrumental variable *[32, 39, 40]. An instrumental variable is a measured factor related to treatment choice but is assumed to be related to study outcomes only through its impact on treatment choice and has no association with unmeasured confounders [39, 40]. Estimates from traditional instrument variable estimators like two-stage least squares (2SLS) have known properties with respect to strength of the instrument to influence treatment choice [41–43] and have distinct estimate interpretations that are especially important with treatment effect heterogeneity [44–48]. While IV-CFA has the potential to provide personalized treatment effect evidence using observational data, the consistency and interpretation of the personalized evidence based on IV-CFA estimates with respect to the pre-specified modeling parameters within the algorithm remain unclear. Estimates from IV-CFA will be more useful for personalized evidence they are not dependent on the parameters of the algorithm.

In this paper we investigate the consistency of IV-CFA personalized treatment effect evidence produced when varying the key algorithm parameters through application to an existing empirical database that revealed treatment effect heterogeneity using instrumental variable estimators with a strong instrumental variable [49]. The database is from an instrumental variable (IV) study for Medicare fee-for-service patients in 2011 that assessed the effects of early surgery on patients with new proximal humerus fractures (PHF) [49]. Meta-analyses indicate that the benefits and detriments of early surgery relative to conservative management are likely heterogeneous across PHF patients [50] but consensus has not been reached on which patients are good candidates for early surgery [51–54]. It is thought that 15–30% of elderly PHF patients are good candidates for early surgery [55–58]. The benefits of early surgery on pain and function are thought to increase with fracture complexity (i.e. extent of displacement, number of fracture parts) [50] and the risk of detriment from early surgery are thought to increase with fracture complexity, patient age, number of comorbidities, frailty, and social independence [58–60]. The prior study used local area surgery rates as an instrumental variable and revealed positive associations between early surgery rates and rates of detrimental outcomes (1-year mortality and morbidity rates). In addition, early surgery effects on detriments varied when patients were stratified a priori by single baseline factors [49]. It remains unknown whether additional surgery effect heterogeneity exists across the population and the extent to which early surgery benefits patients.

With this database we explored the consistency of IV-CFA estimates to provide personalized evidence using two steps. In the first step, we contrasted the distributions of individual early surgery effect estimates across the study population produced using different combinations of IV-CFA parameters. In the second step we applied standard classification and regression trees (CART) to the IV-CFA estimates from the first step to stratify patients into *ex-post reference classes* and assessed the consistency of ex-post reference classes to variation in IV-CFA parameters. In addition, for a representative IV-CFA parameter combination as suggested in the literture [34, 61], we applied two-stage least squares (2SLS) estimators to the patients in the ex-post reference classes to estimate the effects of early surgery on study outcomes and interpret the estimates in terms of known 2SLS properties [41–48].

## Methods

### Population

We used data for Medicare fee-for-service patients with a new proximal humerus fracture (PHF) in 2011 [49]. The prior study included 72,823 patients [49] and used a measure of local area surgical practice styles as an instrument (see the description below). As recommended by the literature [62], additional control variables were specified for this type of instrument including county-level life expectancy [63] and county-level adjusted per capita Medicare spending [64]. Inconsistent links between county identifiers across data sources reduced the population in this study to 72,751 patients.

### Measures

#### Instrumental variable

Health services researchers across clinical areas have noted surgery rates varying dramatically across geographic areas independent of measured differences in patient characteristics and have labeled this phenomena as local area “surgical signatures” [65–70]. It is theorized that providers in a local areas develop “idiosyncratic clinical rules of thumb” leading to these signatures [68, 69]. Local area treatment signatures have been a rich and practical basis for natural experiments [40] in treatment choice across clinical areas [25, 49, 71–89]. Uses of local area treatment signatures to provide natural experiments in treatment choice are based on the assumption that the distributions of unmeasured patient characteristics that affect outcomes across local areas are independent of local area clinical rules of thumb. The prior study used risk-adjusted area surgery ratios (ASRs) in the use of early surgery for PHF patients for each Hospital Referral Region (HRRs) as a measure of local area surgical practice style [49]. An ASR was calculated for each HRR as the ratio of the number of PHF patients in the HRR who received early surgery over the sum of predicted probabilities across the patients in the HRR to receive early surgery. Logistic regression estimates of early surgery choice as a function of measured patient baseline factors (listed in the Reference Class Factors section below) over the study sample was used to estimate the predicted probability of receiving early surgery for each patient. Patients were assigned the ASR value of their resident HRR as the instrumental variable. This instrumental variable provides a natural experiment in early surgery choice under the assumption that ASR variation across HRRs reflects mainly differences in surgeon practice-styles across HRRs and not differences in the distributions of unmeasured patient characteristics like fracture complexity. This assumption is based on the notion that patient residency decisions made years prior to a PHF are likely unrelated to future PHF complexity. Previous research suggests that the bias risk associated with differences in unmeasured patient characteristics is attenuated when larger geographic areas such as the HRRs are used [88]. Nevertheless, this remains an assumption underlying our estimates [88].

#### Treatment

PHF patients who received either reverse shoulder arthroplasty, hemiarthroplasty, open reduction internal fixation, or closed reduction internal fixation) during the 60 days following the PHF index visit were classified as early surgery patients. Surgery claims were identified using Medicare Part B carrier, outpatient, and inpatient claims files.

#### Outcomes

Early surgery for PHF can benefit patients relative to conservative care through increased mobility and reduced pain but can also increase the risk of detriments including death and adverse events [49, 90]. Accordingly, a detriment outcome variable was set equal to 1 if the patient died or had an adverse event during the period 61–365 days following the index PHF, 0 otherwise. Adverse events were measured using Part A and B Medicare claims using ICD-9 codes listed in the prior research [49]. Death was measured using death dates from the 2011 and 2012 Medicare Beneficiary Summary File. In addition, we calculated an “event-based [71] or “process of recovery” [72] measure of benefit for each patient [71–81]. Continued shoulder treatment in the outcome period suggests that a patient had either not fully alleviated pain or not returned to normal function. Our clinical coinvestigators advised that PHF patients progressing toward full pain alleviation and normal function after treatment may still have as many as four evaluation and management (E&M) visits with a surgeon or physical therapist during the period 61–365 days following the index PHF. We estimated the average E&M Medicare cost per shoulder-related visit around $75 in 2011. Accordingly, for each patient a benefit outcome variable was set equal to 1 if the patient survived the period 61–365 days after index PHF with less than $300 of shoulder-related healthcare costs during this period, 0 otherwise. Medicare Part A and Part B claims during the outcome period with one of 192 ICD-9 shoulder diagnoses described in the prior paper were deemed to be shoulder-related [49].

#### Reference class factors

Baseline factors for the IV-CFA algorithm and reference class creation were the patient baseline factors used in the prior study: patient age grouped as 66–69, 70–75, 76–79, 80–85, 86 + , sex, race, Medicaid dual eligibility, and previous shoulder diagnoses of osteoarthritis, rheumatoid arthritis, rotator cuff arthropathy, and avascular necrosis. Medicare claims in the year prior to the index PHF were used to estimate the Charlson Comorbidity Index (CCI) [91, 92], the Frailty Risk Index (FRI) score [93], and quintiles of patient-specific total Medicare spending in the year prior to index [52, 94–96]. Prior healthcare spending has been shown to be indicative of patient health status and health care utilization preferences [52, 95, 96]. For the IV-CFA, the five age groups, CCI, FRI, and the prior health cost quintiles were each specified as single ordinal index variables so that concept “cut-offs” produced by IV-CFA implied monotonic relationships (e.g. a cut-off of less than or equal to age group 3 implies all patients age less than or equal to 79, versus 80 and above). In 2SLS estimation, the baseline factors which were free to vary within a reference class were specified using binary variables for each level of the concept. It should be noted that Medicare data has limited ability to measure certain factors suggested by the literature to affect early surgery effectiveness across patients. Of significant note, the International Classification of Diseases, Ninth Revision (ICD-9) diagnosis codes used in 2011 do not differentiate PHFs by fracture complexity and our results must be interpreted accordingly.

### Empirical approach

Causal forest algorithms (CFAs) evolved from standard classification and regression trees (CART) and random forest ensemble methods [32, 35–38]. CART predictive modeling procedures iteratively partition “nodes” of observations of a study sample into subgroups or sub-nodes based on values of measured baseline factors in a manner which maximizes the differences in an outcome across sub-nodes[37]. A tree is formed by viewing the partitions as “branches” from the full study population into the sub-nodes. The final sub-nodes in a tree are referred to as “leaves”. Minimum “leaf size” parameters in terms of number of observations are available in CART algorithms to stop the branching process. The random forest approach is an ensemble method for prediction which generates a “forest” of CART trees through resampling from the underlying population [36]. The predicted outcome for each patient in a study population is the average outcome across the leaves in the trees in the forest containing the patient. The number of trees in a forest is also a parameter to be specified in random forest algorithms. CFAs extend the random forest approach to the goal of estimating the causal effect of a predictor of interest (e.g. a treatment) on an outcome. CFAs partition observations based on baseline factors which maximize the expected differences *in the estimated treatment effect* on an outcome [32, 35, 38]. When applied to observational data, CFAs suffer the same bias risk from unmeasured confounding that afflicts standard regression. To reduce confounding risk, IV-CFA partitions patients into causal trees using baseline factor combinations which maximize the differences in estimated treatment effect on an outcome *using only the treatment variation associated with an instrumental variable *[32, 39, 40]. For each tree in a forest, IV-CFA assigns to each patient in the population the estimated treatment effect for the leaf on the tree that matches the patient’s baseline factor values. The final estimate of the treatment effect for each patient equals the average estimated effect across the trees in the forest.

In this study we applied IV-CFA to the population of PHF patients and estimated separate models for detriment and benefit outcomes. IV-CFA was implemented using the “grf” package in R [97]. This package provides parameters to vary the number of trees in a forest and the minimum population leaf size in each tree. For each outcome we repeated estimation by varying the forest size and minimum leaf size parameters. Models were run using combinations of either 3000, 4000 or 5000 trees to support large sample properties and minimum leaf sizes of 50, 100, 200, 300, or 400 observations. CFA studies have suggested that larger minimum leaf sizes are needed to avoid over-fitting the models but there is little additional guidance available as to the inferential tradeoffs associated with this parameter choice [98–100]. All IV-CFA scenarios were estimated using the “honest” approach suggested by the algorithm creators in which each tree was estimated using a randomly selected 25% of the study population [38]. To assess the consistency of estimates across algorithm parameters we report the distribution of estimated surgery effects for both the detriment and benefit outcomes by combinations of the parameters.

In the second step, we use standard classification and regression trees (CART) to stratify patients into *ex-post reference classes *based on the early surgery effect estimates from IV-CFA in the first step using baseline patient factors and assessed the consistency of these ex-post reference classes to variation in IV-CFA model parameters. In addition, for a representative IV-CFA model parameter combination, we applied two-stage least squares (2SLS) estimators to the patients in each ex-post reference class to estimate the effects of early surgery on each study outcome and interpret the estimates from each ex-post reference class in terms of known 2SLS properties [41–48]. In contrast to the IV-CFA estimator, the 2SLS estimator statistically controls for the remaining baseline factors not used in creating the reference class. Instrumental variable estimators require strong relationships between treatment choice and the specified instrument to yield valid results [101]. 2SLS provides an F-statistic assessing the strength of this relationship between the instrument from the 1^st^stage of 2SLS and early surgery choice, whereas IV-CFA does not provide such evidence. 2SLS estimates a local average treatment effect (LATE) for the subset of patients within each ex-post reference class whose early surgery choice were sensitive to the instrumental variable [22, 25, 26, 43, 102–106]. Early surgery effects will remain heterogeneous across patients within each ex-post reference class if the baseline factors affecting early surgery effectiveness are incompletely measured in an empirical database. If early surgery choice within an ex-post reference class reflects these unmeasured factors (what is known as *essential heterogeneity* or sorting on the gain), estimates will not generalize to all patients within an ex-post reference class and must be properly interpreted [22, 26, 44–48]. To gain insight into these inferential distinctions, for each ex-post reference class we provide the percentage of patients who received early surgery, the range of early-surgery rates across patients grouped by the quintiles of the instrument, the 1^st^ stage F-statistic, and the 2SLS-estimated effect of early surgery on the probability of patients attaining the respective outcome. 2SLS estimates are displayed in 2-way tables showing the ex-post reference classes for both benefit and detriment outcomes to enable decision-makers to find evidence appropriately personalized (to the extent possible) for a new patient.

## Results

For context, Tables A.1 and A.2 in the Additional file 1 reproduces the format of the patient factor summary tables found in the prior research for this study population [49]. Table A.1 groups patients by early surgery choice and Table A.2 distributes patients across quintiles of local area early surgery ratios (ASRs). Early surgery patients in Table A.1 were more likely to have a detriment outcome and less likely to have a benefit outcome than conservatively managed patients. In Table A.2, higher early surgery rates across ASR quintiles were also associated with higher probabilities of the detriment outcome, but in contrast to Table A.1, higher early surgery rates across ASR quintiles were associated with *higher* probabilities of the benefit outcome.

Tables 1 and 2 show summary statistics of the distributions of IV-CFA estimated effects of early surgery relative to conservative management across the study population by combinations of algorithm parameters for the benefit and detriment outcomes, respectively. IV-CFA yields estimates of the absolute effect of early surgery on the probability of the specified outcome occurring for each patient. For example, the estimated absolute effect of early surgery on the benefit outcome using 4000 trees with a minimum node size of 200 patients for the patient at the 75% percentile in Table 1 is 0.312. This means that IV-CFA estimates that early surgery increases the probability of the benefit outcome for that patient by 31.2%. Likewise, with 4000 trees and a minimum node size of 200 patients in Table 2, 2-CFA estimates that early surgery increases the probability of the detriment outcome for the patient at the 25% percentile in that distribution by 4.7%. With 4000 trees and a minimum node size of 200 patients the inter-decile range of early surgery effect was (-0.038 to 0.421) for benefit and (-0.017 to 0.283) for detriment which suggests substantial heterogeneity in early surgery effects across the study population for each outcome. Tables 1 and 2 show that the average absolute early surgery effects (mean and median) remain consistent across minimum leaf sizes for both outcomes, but the ranges of the estimates across the population increase substantially as the minimum leaf size falls from 400 to 50. Using 4000 trees, the inter-decile range of early surgery effect on the benefit outcome with a minimum leaf size of 400 is (0.031 to 0.236) in contrast to (-0.188 to 0.462) with a minimum leaf size of 50. In addition, for each patient we estimated the range of early surgery effect estimates across the minimum leaf size values (50,100,200,300,400) for 4000 trees. Over half of the patients had early surgery effect estimate ranges across these minimum leaf sizes of more than 0.150 for the benefit outcome and 0.120 for the detriment outcome. These range estimates are large when compared to the mean estimated effects of early surgery on the benefit outcome (0.196) and the detriment outcome (0.135) across the population. Increasing the number of trees in each IV-CFA forest lowered the standard deviation of mean outcomes but had no appreciable effects on the distribution of estimated effects across the population.

**Table 1:** Distribution of Instrumental Variable Causal Forest Algorithm Early Surgery Absolute Effects on the Benefit Outcome^a^ for Medicare Patients with Proximal Humerus Fractures in 2011 by Number of Trees in IV-CFA Forest and Minimum Leaf Node Population Size in Each Tree

Trees in IV-CFA Forest	Minimum Leaf Node Population Size	Mean	St Dev	Percent of Patients with Positive Effect	Min	10^th^	25^th^	50^th^ Median	75^th^	90^th^	Max
3000	50	.198	.327	75%	-1.448	-.193	.0001	.203	.411	.586	1.368
	100	.196	.236	81%	-.729	-.088	.056	.204	.350	.481	.908
	200	.197	.175	88%	-.449	-.033	.100	.210	.311	.418	.675
	300	.198	.147	90%	-.284	-.005	.110	.213	.299	.373	.587
	400	.198	.131	91%	-.220	.013	.118	.214	.290	.358	.518
4000	50	.197	.331	75%	-1.457	-.208	-.004	.200	.413	.593	1.366
	100	.197	.237	81%	-.727	-.094	.058	.204	.353	.480	.906
	200	.196	.177	87%	-.434	-.038	.096	.208	.312	.421	.659
	300	.197	.148	90%	-.283	-.008	.109	.213	.299	.374	.565
	400	.198	.132	91%	-.206	.014	.116	.215	.290	.362	.512
5000	50	.198	.327	75%	-1.389	-.201	.003	.202	.413	.590	1.375
	100	.197	.234	81%	-.721	-.087	.060	.201	.352	.477	.882
	200	.197	.172	87%	-.417	-.032	.100	.209	.308	.414	.663
	300	.198	.145	90%	-.278	-.004	.112	.214	.296	.369	.560
	400	.198	.129	91%	-.189	.018	.117	.217	.286	.354	.505

^a^1 if patient survives 61–365 days after index proximal humerus fracture with less than $300 of shoulder-related healthcare costs, 0 otherwise

**Table 2:** Distribution of Instrumental Variable Causal Forest Algorithm Early Surgery Absolute Effects on the **Detriment** Outcome^a^ for Medicare Patients with Proximal Humerus Fractures in 2011 by Number of Trees in IV-CFA Forest and Minimum Leaf Node Population Size in Each Tree

Trees in IV-CFA Forest	Minimum Leaf Node Population Size	Mean	St Dev	Percent of Patients with Positive Effect	Min	10^th^	25^th^	50^th^ Median	75^th^	90^th^	Max
3000	50	.139	.265	72%	-1.369	-.186	-.021	.143	.304	.468	1.107
	100	.137	.172	77%	-.376	-.086	.013	.138	.254	.354	.679
	200	.137	.115	87%	-.207	-.014	.048	.146	.218	.288	.457
	300	.136	.093	92%	-.158	.012	.067	.141	.202	.255	.383
	400	.137	.077	96%	-.105	.032	.082	.143	.191	.239	.351
4000	50	.135	.264	72%	-1.254	-.188	-.020	.136	.297	.462	1.090
	100	.136	.171	77%	-.357	-.086	.015	.136	.251	.350	.703
	200	.135	.115	86%	-.200	-.017	.047	.142	.219	.283	.430
	300	.136	.093	92%	-.145	.009	.067	.142	.203	.256	.373
	400	.135	.078	95%	-.106	.031	.081	.140	.190	.236	.338
5000	50	.136	.263	71%	-1.209	-.189	-.023	.139	.300	.462	1.152
	100	.137	.171	77%	-.381	-.081	.013	.136	.251	.350	.697
	200	.138	.114	87%	-.194	-.014	.048	.144	.218	.286	.435
	300	.136	.091	93%	-.142	.012	.069	.142	.202	.254	.384
	400	.136	.078	95%	-.089	.031	.080	.141	.191	.237	.350

^a^1 the patient died or had an adverse event during the period 61–365 days following the index PHF, 0 otherwise

**Fig. 1:**
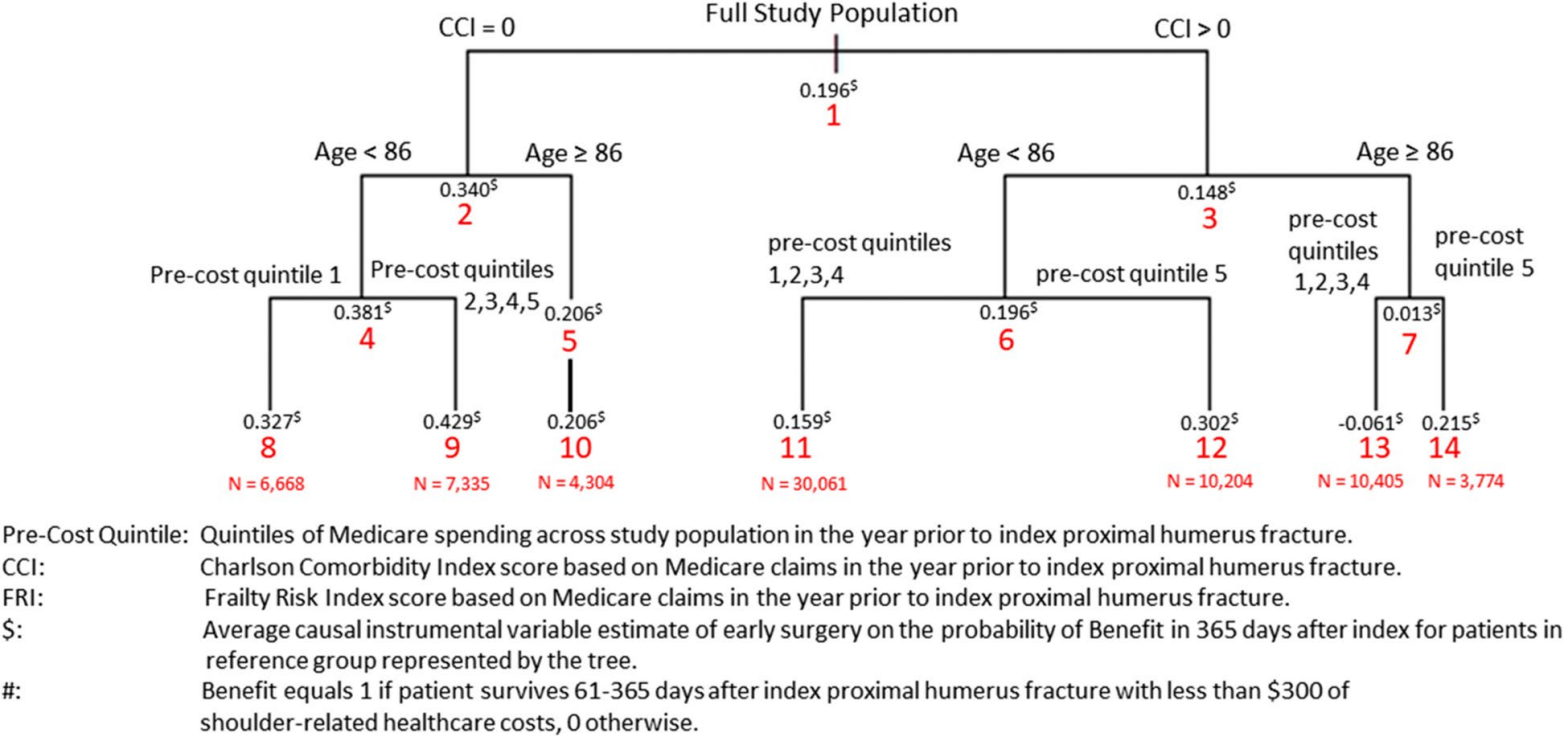
Regression Tree for Causal Instrumental Variable-Based Early Surgery Effect on the Probability of Benefit^#^

**Fig. 2:**
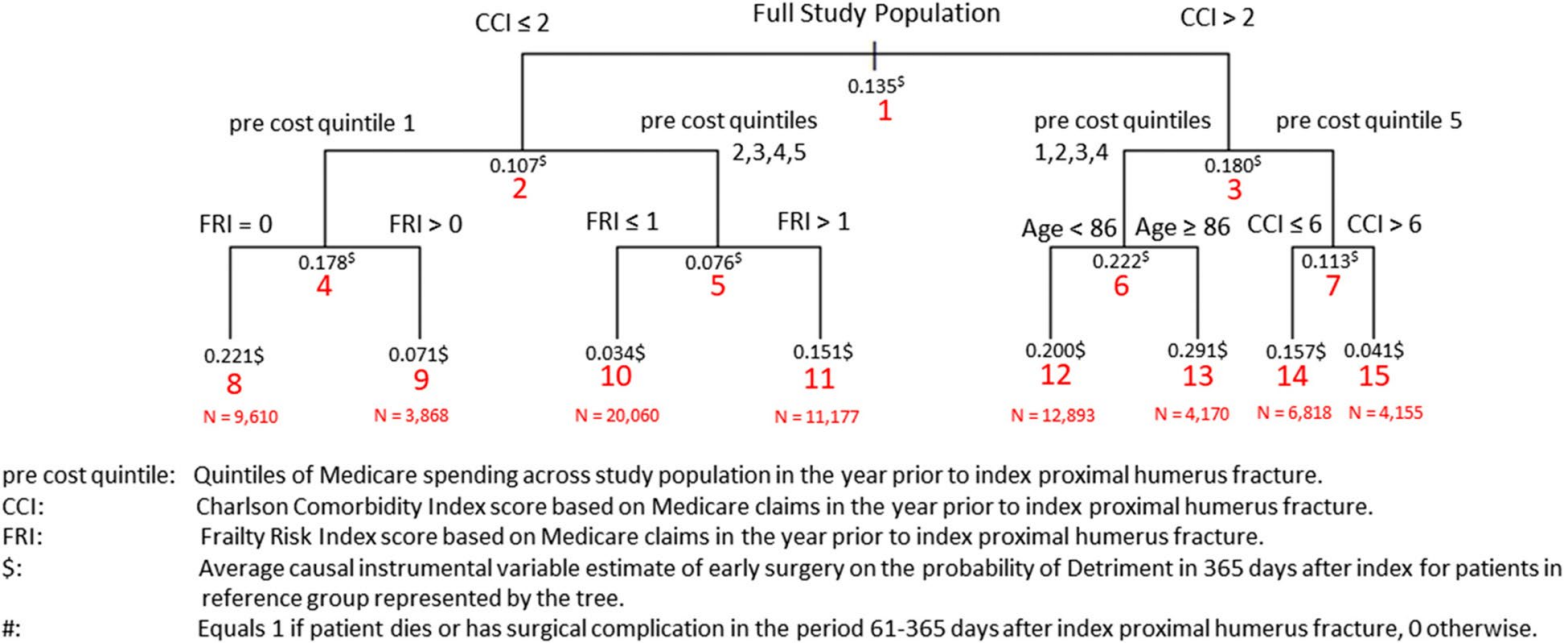
Regression Tree for Causal Instrumental Variable-Based Early Surgery Effect on the Probability of Detriment^#^

In the second step, we assessed the consistency of ex-post reference classes derived from the IV-CFA estimates in the first step using standard classification and regression trees (CART). Because the number of trees had no appreciable effects on the estimates in Tables 1 and 2, we focused on scenarios using 4000 trees and varied the minimum leaf size in IV-CFA. To illustrate this approach, Figs. 1 and 2 show the CART results using the minimum leaf size of 200 on the benefit and detriment outcomes, respectively. The values above the red node label in each figure equal the average IV-CFA early surgery absolute effect estimate on the respective outcome for the patients in that node. Node 1 in both figures contains estimates for the full population with values consistent with the means in Tables 1 and 2. For the benefit outcome in Fig. 1, CART divided the population initially by whether patients had a CCI score of 0 (node 2) or greater than zero (node 3). The average IV-CFA estimate of early surgery effect on the benefit outcome in node 2 (0.340) was nearly 130% higher than in node 3 (0.148). Age < 86 caused the second level of splits (nodes 4–7) with average early surgery benefits higher for the patients under 86. In the third-level of splits (nodes 8–14), patients with higher levels of Medicare spending prior to a PHF had higher probabilities of benefiting from early surgery. The estimated average absolute effect of early surgery on the benefit outcome for the ex-post reference classes in the third-level split in Fig. 1 ranged from a low of (-0.061) in node 13 to (0.429) in node 9. Figure 2 displays the CART result for the effects of early surgery on the detriment outcome. Patients with higher CCI, lower pre-PHF Medicare spending, older age, and higher FRI scores were more likely to have a detrimental outcome from early surgery. The estimated effect of early surgery for the ex-post reference classes on the detriment outcome in the third-level split in Fig. 2 ranged from a low of (0.034) in node 10 to (0.291) in node 13. This approach isolated ex-post reference groups with combinations of baseline patient factors which showed distinct levels early surgery effects for both detriment and benefit outcomes which were not identified in the previous research that specified reference classes a priori [49].

Tables 3 and 4 describe the ex-post reference classes in terms of the patient baseline factors used to construct the third-level CART nodes for the minimum leaf sizes in Tables 1 and 2 for the benefit and detriment outcomes, respectively. In Table 3 the third-level nodes are sorted from left to right from the highest early surgery effect on the benefit outcome to the least. In Table 4 the third-level nodes are sorted from left to right from the least early surgery effect on the detriment outcome to most. Despite the broad variation in the ranges of early surgery effect estimates in Tables 1 and 2 across minimum leaf sizes, the patient baseline factors used by CART to construct ex-post reference classes (CCI, age, pre-fracture Medicare spending, FRI) were consistent across minimum leaf sizes. For the detriment outcome in Table 4, the ex-post reference class definitions are identical for minimum leaf sizes greater than 100 with only the effect order differing for the first two nodes at minimum leaf size of 100. For the benefit outcome in Table 3, the ex-post reference classes are consistent from minimum leaf sizes greater that 200 with only a difference in benefit effect ordering across ex-post reference classes moving from 200 to 300 minimum leaf sizes.

**Table 3:** Third-level Split Ex-Post Reference Class Designations by Minimum Leaf Node Size in IV-CFA Estimation on the Benefit Outcome for Medicare Patients with Proximal Humerus Fractures in 2011 with 4000 Trees in the Forest

	Third-level Split Nodes							
Minimum Leaf Node Size	Highest ← Early Surgery Effect on Benefit Outcome → Lowest							
50	< 86, CCI = 0, Not in lowest 2 pre cost quintiles	≥ 86, Highest pre cost quintile, CCI > 4	< 86, CCI = 0, Lowest 2 pre cost quintiles	< 86, CCI > 0, Highest pre cost quintile	< 86, CCI > 0, Not highest pre cost quintile	≥ 86, Highest pre cost quintile, CCI ≤ 4	≥ 86, Not highest pre cost quintile, FRI ≤ 2	≥ 86, Not highest pre cost quintile, FRI > 2
100	< 86, CCI = 0, Not in lowest pre cost quintile	≥ 86, Highest pre cost quintile, CCI > 4	< 86, CCI = 0, Lowest pre cost quintile	< 86, CCI > 0, Highest pre cost quintile	< 86, CCI > 0, Not highest pre cost quintile	≥ 86, Not highest pre cost quintile, CCI ≤ 1	≥ 86, Highest pre cost quintile, CCI ≤ 4	≥ 86, Not highest pre cost quintile, CCI > 1
200	CCI = 0, < 86, Not in lowest pre cost quintile	CCI = 0, < 86, Lowest pre cost quintile	CCI > 0, < 86, Highest pre cost quintile	CCI > 0, ≥ 86, Highest pre cost quintile	CCI = 0, ≥ 86	CCI = 0, ≥ 86	CCI > 0, < 86, Not highest pre cost quintile	CCI > 0, ≥ 86, Not highest pre cost quintile
300	CCI = 0, < 86, Not in lowest pre cost quintile	CCI = 0, < 86, Lowest pre cost quintile	CCI > 0, < 86, Highest pre cost quintile	CCI = 0, ≥ 86	CCI = 0, ≥ 86	CCI > 0, ≥ 86, Highest pre cost quintile	CCI > 0, < 86, Not highest pre cost quintile	CCI > 0, ≥ 86, Not highest pre cost quintile
400	CCI = 0, < 86, Not in lowest pre cost quintile	CCI > 4, Highest pre cost quintile	CCI = 0, < 86, Lowest pre cost quintile	CCI = 0, ≥ 86	CCI = 0, ≥ 86	0 < CCI ≤ 4, Highest pre cost quintile	CCI > 0, < 86, Not highest pre cost quintile	CCI > 0, ≥ 86, Not highest pre cost quintile

CCI: Charlson Comorbidity Index score based on Medicare claims in the year prior to index proximal humerus fracture

FRI: Frailty Risk Index score based on Medicare claims in the year prior to index proximal humerus fracture

Cost Quintiles: Based on Medicare spending in the 365 days prior to the index PHF

Benefit: 1 if patient survives 61–365 days after index proximal humerus fracture with less than $300 of shoulder-related healthcare costs, 0 otherwise

**Table 4:** Third-level Split Ex-Post Reference Class Designations by Minimum Leaf Node Size in IV-CFA Estimation on the **Detriment** Outcome for Medicare Patients with Proximal Humerus Fractures in 2011 with 4000 Trees in the Forest

	Third-level Split Nodes							
Minimum Leaf Size	Lowest ← Early Surgery Effect on Detriment Outcome → Highest							
50	CCI ≤ 2, Lowest pre cost quintile, FRI > 0	CCI ≤ 2, Not lowest pre cost quintile, FRI ≤ 1	CCI > 6, Highest pre cost quintile	CCI ≤ 2, Not lowest pre cost quintile, FRI > 1	CCI > 6, Not highest pre cost quintile	2 < CCI ≤ 6, 70 +	CCI ≤ 2, Lowest pre cost quintile, FRI = 0	2 < CCI ≤ 6, < 70
100	CCI > 6, Highest pre cost quintile	CCI ≤ 2, Not lowest pre cost quintile, FRI ≤ 1	CCI ≤ 2, Lowest pre cost quintile, FRI > 0	CCI ≤ 2, Not lowest pre cost quintile, FRI > 1	2 < CCI ≤ 6, Highest pre cost quintile	CCI > 2, Not highest pre cost quintile, < 86	CCI ≤ 2, Lowest pre cost quintile, FRI = 0	CCI > 2, Not highest pre cost quintile, 86 +
200	CCI ≤ 2, Not lowest pre cost quintile, FRI ≤ 1	CCI > 6, Highest pre cost quintile	CCI ≤ 2, Lowest pre cost quintile, FRI > 0	CCI ≤ 2, Not lowest pre cost quintile, FRI > 1	2 < CCI ≤ 6, Highest pre cost quintile	CCI > 2, Not highest pre cost quintile, < 86	CCI ≤ 2, Lowest pre cost quintile, FRI = 0	CCI > 2, Not highest pre cost quintile, 86 +
300	CCI ≤ 2, Not lowest pre cost quintile, FRI ≤ 1	CCI > 6, Highest pre cost quintile	CCI ≤ 2, Lowest pre cost quintile, FRI > 0	CCI ≤ 2, Not lowest pre cost quintile, FRI > 1	2 < CCI ≤ 6, Highest pre cost quintile	CCI > 2, Not highest pre cost quintile, < 86	CCI ≤ 2, Lowest pre cost quintile, FRI = 0	CCI > 2, Not highest pre cost quintile, 86 +
400	CCI ≤ 2, Not lowest pre cost quintile, FRI ≤ 1	CCI > 6, Highest pre cost quintile	CCI ≤ 2, Lowest pre cost quintile, FRI > 0	CCI ≤ 2, Not lowest pre cost quintile, FRI > 1	2 < CCI ≤ 6, Highest pre cost quintile	CCI > 2, Not highest pre cost quintile, < 86	CCI ≤ 2, Lowest pre cost quintile, FRI = 0	CCI > 2, Not highest pre cost quintile, 86 +

CCI: Charlson Comorbidity Index score based on Medicare claims in the year prior to index proximal humerus fracture

FRI: Frailty Risk Index score based on Medicare claims in the year prior to index proximal humerus fracture

Cost Quintiles: Based on Medicare spending in the 365 days prior to the index PHF

Detriment: 1 the patient died or had an adverse event during the period 61–365 days following the index PHF, 0 otherwise. Adverse events include pneumonia, cardiac dysrhythmias, congestive heart failure, deep vein thrombosis or pulmonary embolism, infection, nerve injury, prosthetic complication, hematoma, avascular necrosis, adhesive capsulitis, and instability or dislocation

Table 5 summarizes 2SLS estimates for the ex-post reference classes at the third-level splits found in Figs. 1 and 2 using IV-CFA parameters of 4000 trees and minimum leaf size of 200. The 2SLS early surgery effect estimates on the benefit outcome for the ex-post reference classes in nodes 8–14 in Fig. 1 are described in the rows and the 2SLS early surgery effect estimates on the detriment outcome for the ex-post reference classes in nodes 8–15 in Fig. 2are described in the columns. The rows are arranged with the estimated effects of early surgery on the benefit outcome for each ex-post reference class decreasing from top to bottom. The right-most column summarizes the 2SLS estimates for the ex-post reference class represented by each row, including the early-surgery rate (R), the interquartile range of early surgery rate across local areas defined by Hospital Referral Regions (HRRs), the F-statistic of the effect of the instrument on early-surgery choice in the first stage of 2SLS, the 2SLS estimated absolute effect of early surgery on the benefit outcome (IVE), and the number of patients in the ex-post reference class (N). Likewise, the detriment ex-post reference classes are represented in the columns and are arranged with the estimated effects of early surgery on the detriment outcome for each ex-post reference class increasing moving from left to right. The bottom row summarizes the 2SLS estimates for the ex-post reference class represented by each column. The “southeast” cell in Table 5 provides the 2SLS results for the full study population with the absolute effect of early surgery on the benefit outcome (B_IVE) and the absolute effect of early surgery on the detriment outcome (D_IVE). Following 2SLS literature [22, 25, 26, 43, 102–106], the 2SLS estimates in this table represent the local average early surgery effect on the respective outcome for the PHF patients in each ex-post reference class whose early surgery choices were sensitive to the instrument value. 2SLS estimation directly controls for the baseline factors free to vary within each ex-post reference class, so that these estimates differ from the IV-CFA estimates in Figs. 1 and 2which do not control for these factors. For each benefit and detriment ex-post reference class in Table 5, the instrument had a statistically significant “non-weak” (F statistic greater than 10) effect on early surgery choice [101]. Early surgery effect heterogeneity is apparent for both outcomes across ex-post reference classes. The 2SLS estimated absolute effects of early surgery on benefit outcome varied from (0.589) in ex-post reference class 9 to (-0.323) in ex-post reference class 13. The 2SLS estimated absolute effect of early surgery on detriment varied from (0.677) in detriment ex-post reference class 13 to (-0.291) in detriment ex-post reference class 15.

**Table 5:**
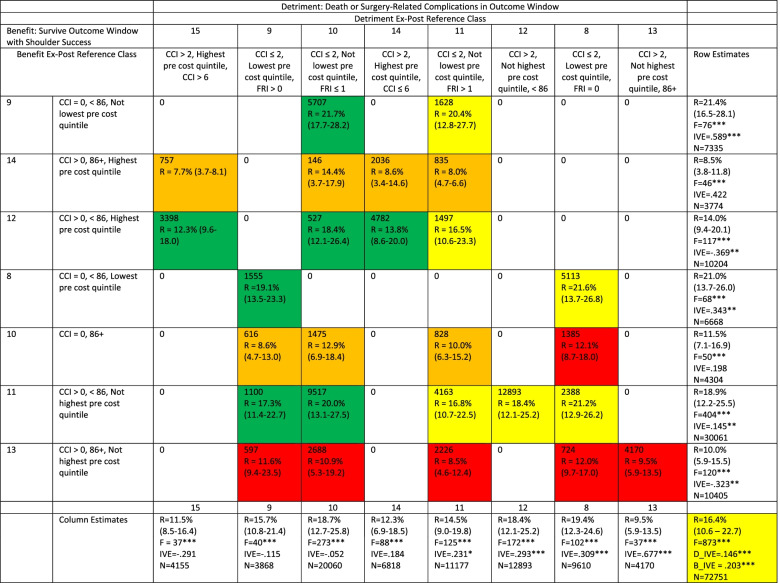
Two-Stage Least Squares (2SLS) Estimates by Benefit and Detriment by Ex-Post Reference Classes for Third-level CART Splits

R = early surgery rate; (*,*) = the inter-quintile range in early surgery rates across patients in the node; F = F-statistic of the effect of the instrument on early surgery choice in the first stage; IVE = the 2SLS estimated absolute effect of early surgery on the probability of the respective outcome; N = the number of patients in the node; D_IVE the study population wide 2SLS absolute effect of early surgery on detriment; and B_IVE the study population wide 2SLS absolute effect of early surgery on benefit. CCI: Charlson Comorbidity Index score based on Medicare claims in the year prior to index proximal humerus fracture. FRI: Frailty Risk Index score based on Medicare claims in the year prior to index proximal humerus fracture

^*^, **, *** *p* < .1, *p* < .05, *p* < .01 respectively

It should be noted that the ex-post reference classes found using this approach for the benefit and detriment outcomes are different and the cells within Table 5 describe the patients represented by the intersection of the respective benefit and detriment ex-post reference classes including number of patients, the early surgery rate, and the interquartile range in early surgery rates across local areas for those patients. Green cells contain patients with a positive and statistically significant benefit increase from higher rates of early surgery and no statistically significant detriment increase from higher early surgery rates. Red cells contain patients with no statistically significant benefit increase from higher early surgery rates and a statistically significant detriment increase from higher early surgery rates. Yellow cells contain patients with a statistically significant benefit increase from higher early surgery rates and a statistically significant detriment increase from higher early surgery rates. Orange cells contain patients no statistically significant benefit increase from higher early surgery rates and no statistically significant estimated detriment increase from higher early surgery rates. Note that the overall study population is a yellow cell as higher early surgery rates across the full population are associated with both higher rates of benefit and detriment outcomes.

## Discussion

With acknowledged treatment effect heterogeneity [4–10], getting *personalized evidence* to patients is a policy priority [1–3].Randomized controlled trials cannot generate personalized evidence for many patients [4, 11–14]. The use of comparative effectiveness research (CER) methods to exploit treatment variation in the diverse patients in large observational databases has been offered as an alternative [1–3, 11, 15–18]. With traditional CER methods, finding personalized evidence involves aligning a patient with “reference class” of patients using baseline factors *specified prior to estimation *and summarizing treatment effect evidence for those patients [19–21]. IV-CFA was developed to directly estimate patient-specific treatment effects within a study population using observational data based on measured baseline patient factors without having to specify a reference class a prioir [32]. To avoid confounding bias, IV-CFA uses treatment variation only from a specified instrumental variable. However, IV-CFA estimates are conditional on parameters in the algorithm. For a population of Medicare beneficiaries with proximal humerus fractures we assessed the usefulness of IV-CFA to generate personalized evidence by varying key algorithm parameters and assessing (1) the consistency of individual IV-CFA estimates of early surgery effects on benefit and detriment outcomes and (2) the consistency of ex-post reference classes produced from a CART procedure that grouped patients with similar IV-CFA estimated effects from early surgery. We then applied 2SLS instrumental variable estimator to the patients within representative ex-post reference classes and interpreted results with respect to known 2SLS properties.

The study population consisted of Medicare beneficiaries with new proximal humerus fractures (PHFs) in 2011 from an earlier study which showed heterogeneity in effect of early surgery on detriments based on reference classes specified prior to analyses [49]. With its large sample size and a “strong” instrumental variable, this database supplied a solid foundation to assess the properties of the IV-CFA approach to generate consistent personalized evidence on the effects of early surgery across outcomes. It should be noted that fracture complexity is a recognized source of early surgery effect heterogeneity for this population [58–60], and complexity could not be measured using Medicare claims in 2011. The instrumental variable approach used in this study assumes that the distributions of fracture complexity across PHF patients are similar across HRRs. However, with fracture complexity unmeasured for each patient, we can state, a priori, that if early surgery choice reflects unmeasured factor complexity which affects early surgery effectiveness (what is known as *essential heterogeneity* or sorting on the gain), early surgery effect estimates will not generalize to all patients with the same combination of measured baseline factors and must be properly interpreted [22, 26, 44–48].

IV-CFA estimates showed substantial heterogeneity in the effects of early surgery on both benefit and detriment outcomes across patients with PHF. However, the estimated effects for each PHF patient were conditional on the minimum leaf size used in the algorithm. There is little guidance in the literature or algorithm to support an optimal minimum leaf size. CFA studies suggest that larger minimum leaf sizes are needed to avoid over-fitting the models, but no information exists to designate what is meant by “larger” and the tradeoffs involved with the selection of a minimum leaf size [98–100].

In the second step of our assessment, though, we found that applying a standard CART procedure to the IV-CFA estimates provided ex-post reference classes that remained relatively consistent across the minimum leaf sizes used in IV-CFA. These ex-post reference classes revealed early surgery effect heterogeneity that was not observed in the previously published instrumental variable analyses of these data [49]. 2SLS estimation within each ex-post reference class of patients showed substantial early surgery effect heterogeneity in both detriment and benefit outcomes. The 2SLS estimates for the absolute effect of early surgery for entire sample were (0.146) on the detriment outcome and (0.203) on the benefit outcome as shown in the lower right corner of Table 5. Those overall estimates are very different from the estimates for each ex-post reference class for each outcome. Older PHF patients with more comorbidities, who were frailer, and did use healthcare extensively prior to an index PHF were less likely to have a benefit outcome and more likely to have a detriment outcome from early surgery. As a result, at a minimum, it is clearly inappropriate to generalize estimates from the whole population to many of the ex-post reference classes identified.

However, the 2SLS estimates for each ex-post reference class must be interpreted given the properties of 2SLS estimation and the existence of essential heterogeneity within each ex-post reference class. 2SLS estimates a local average treatment effect (LATE) for the subset of patients within a sample whose treatment choices were sensitive to the instrumental variable [22, 25, 26, 43, 102–106]. With essential heterogeneity, estimates of LATE are best interpreted as evidence of an average treatment effect on an outcome associated with treatment rate changes around the observed treatment rate [22, 26, 43, 103, 105, 107]. Strong additional assumptions are required for LATE estimates to be valid estimates of the average treatment effect across a reference class (ATE), the average treatment effect on the treated in a reference class (ATT), or the average treatment effect on the untreated (ATU) in a reference class [22, 48, 106, 108–110]. For PHF patients, it is well-known that unmeasured fracture complexity impacts the effectiveness of early surgery on benefits and detriments and also likely affects the choice of early surgery. Consequently, 2SLS estimates in Table 6  should be interpreted in the context of early surgery variation associated with the instrumental variable within each ex-post reference class and not in terms of overall effects across all patients within each ex-post reference class. For example, these estimates suggest that higher early surgery rates in the green cells of Table 5 would have increased the benefit probability with little increased detriment risk. In contrast, lower early surgery rates in the red cells would have reduced detriment risk without a benefit loss. Early surgery rate changes in the yellow and orange cells would involve tradeoffs between benefit and detriment changes. With fracture complexity unmeasured for these patients, though, there are likely patients in each ex-post reference class who were perfect candidates for early surgery and perfect candidates for conservative management so that the “right” early surgery rate in each reference class is not likely to be zero or one hundred percent [65, 111–116]. In addition, the estimates within in each ex-post reference class reflect the knowledge and practices in 2011 and the early surgery rates in 2011. If since 2011 early surgery rates in the cells changed, the true LATE for each ex-post reference class will change to reflect the new rates. The dependence of these estimates on the early surgery rates *within* reference classes makes it risky to make comparison estimates *across* reference classes in terms of baseline factors. For example, in Table 5 if the early surgery rate for benefit node 9 in 2011 was closer to the higher quintile for that group (28.1%), the estimated benefits from higher early surgery rates would likely be lower as the additional treated patients in this ex-post reference class would have likely gained less from early surgery. The 2SLS estimated effect of early surgery for node 9 patients would fall in comparison to the other ex-post reference classes without changing the make-up of each class [22].

## Conclusion

In summary, because early surgery effect estimates for individual patients vary with IV-CFA algorithm parameters, the value of using IV-CFA estimates for direct evidence for individuals is questionable. However, applying standard CART procedures to IV-CFA estimates uncovers ex-post reference classes that are robust across ranges in IV-CFA parameters. Because the two-stage least squares (2SLS) estimator yields a local average treatment effect (LATE) for the subset of patients within each ex-post reference class whose early surgery choices were sensitive to the instrumental variable, the extent to which 2SLS estimates can be attributed to individual patients within each ex-post reference class is limited. But these estimates are well-suited to help surgeons assess whether the early surgery rates in their practices for PHF patients within an ex-post reference class reflect over or under utilization. Surgeons with low early surgery rates in green cell ex-post reference classes could feel confident with expending early surgery rates for those patients, and surgeons with higher early surgery rates in red cell reference classes should likely reduce early surgery rates for those patients.

## Supplementary Information

**Additional file 1: Table A.1.**Characteristics of the Study Population by Early Surgery Choice. **Table A.2.** Characteristics of the Study Population by Local Area Early Surgery Ratios.

## Data Availability

The Medicare claims data used here were obtained from ResDAC upon successful application, at https://www.resdac.org.

## Abbreviations

IV-CFA: Instrumental Variable Causal Forest Algorithm
RCTs: Randomized controlled trials
CER: Comparative effectiveness research
CART: Classification and regression trees
CFA: Causal Forest Algorithm
PHF: Proximal Humeral Fracture
CCI: Charlson Comorbidity Index
FRI: Function-Related Indicators
2SLS: Two-stage Least Squares
LATE: Local average treatment effect
ATE: Average treatment effect across a population
ATT: The average treatment effect on the treated in a population
ATU: The average treatment effect on the untreated (ATU) in a population
ResDAC: Research Data Assistance Center
