# Supplementary material for: Assessing the ability of an instrumental variable causal forest algorithm to personalize treatment evidence using observational data: the case of early surgery for shoulder fracture
Source: BMC Med Res Methodol. 2022 Jul 11;22:190. doi: 10.1186/s12874-022-01663-0 (PMC9275148; doi:10.1186/s12874-022-01663-0)
Supplement: Supplementary file 1 — Additional file 1: Table A.1.Characteristics of the Study Population by Early Surgery Choice. Table A.2. Characteristics of the Study Population by Local Area Early Surgery Ratios. [file 12874_2022_1663_MOESM1_ESM.docx]

| Table A.1: Characteristics of the Study Population by Early Surgery Choice | | | | |
| --- | --- | --- | --- | --- |
| Factors | Study Population, (%)  (N = 72,751) | Conservatively Managed, (%)  (N = 60,840) | Early Surgery, (%)  (N = 11,911) | P value§§ |
| **Patient-Specific Factors** |  |  |  |  |
| Male | 19.2 | 19.5 | 17.5 | < 0.001 |
| Age (66-69) | 13.4 | 12.7 | 17.1 | < 0.001 |
| Age (70-75) | 21.1 | 20.1 | 25.8 | < 0.001 |
| Age (76-79) | 15.2 | 14.7 | 17.8 | < 0.001 |
| Age (80-85) | 24.9 | 25.1 | 23.7 | 0.002 |
| Age (86 plus) | 25.4 | 27.3 | 15.5 | < 0.001 |
| Asian | 0.9 | 1.0 | 0.7 | 0.004 |
| Black | 3.1 | 3.3 | 2.0 | < 0.001 |
| Hispanic | 1.4 | 1.4 | 1.0 | 0.001 |
| Other Race | 1.2 | 1.2 | 1.1 | 0.267 |
| White | 93.4 | 93 | 95.1 | < 0.001 |
| Fully Dual Eligible‡‡ | 13.5 | 14.3 | 9.1 | < 0.001 |
| CCI (0) | 25.2 | 24.4 | 29.1 | < 0.001 |
| CCI (1) | 20.7 | 20.5 | 21.7 | 0.003 |
| CCI (2) | 15.6 | 15.7 | 15 | 0.034 |
| CCI (3) | 12.3 | 12.4 | 12.2 | 0.621 |
| CCI (4 plus) | 26.2 | 27.0 | 22.0 | < 0.001 |
| FRI (0) | 35.3 | 34.0 | 41.8 | < 0.001 |
| FRI (1) | 26.3 | 26.1 | 27.1 | 0.027 |
| FRI (2) | 15.7 | 16 | 14.5 | < 0.001 |
| FRI (3 plus) | 22.7 | 23.9 | 16.6 | < 0.001 |
| Medicare Spending Above Mean Prior to Index† | 26.5 | 27.4 | 22.0 | < 0.001 |
| Osteoarthritis | 25.2 | 25.5 | 23.7 | < 0.001 |
| Rheumatoid arthritis | 7.9 | 8.0 | 7.4 | 0.03 |
| Rotator cuff arthropathy | 6.6 | 6.7 | 6.3 | 0.125 |
| Avascular necrosis | 0.2 | 0.1 | 0.3 | 0.011 |
| **County-Level Factors** |  |  |  |  |
| Mean Medicare spending (county) | 9520 | 9518 | 9535 | 0.246 |
| Mean life expectancy (county) | 78.3 | 78.3 | 78.2 | < 0.001 |
| **Outcomes** |  |  |  |  |
| Death (%)‡ | 10.2 | 10.8 | 7.0 | < 0.001 |
| Complication (%)§ | 52.1 | 50.8 | 58.5 | < 0.001 |
| Detriment (%)¶ | 56.8 | 55.7 | 62.9 | < 0.001 |
| Benefit (%)† | 58.3 | 61.2 | 43.2 | < 0.001 |
| CCI: Charlson Comorbidity Index score based on Medicare claims in the year prior to index proximal humerus fracture.  FRI: Frailty Risk Index score based on Medicare claims in the year prior to index proximal humerus fracture.  †: 1 if patient had Medicare spending in the 365 days prior to the index PHF that was greater than the average of patients in the sample, 0 otherwise.  ‡: 1 if patient died during the period 61-365 days following the index PHF, 0 otherwise.  §: 1 if patient had an adverse event during the period 61-365 days following the index PHF, 0 otherwise. Adverse events include pneumonia, cardiac dysrhythmias, congestive heart failure, deep vein thrombosis or pulmonary embolism, infection, nerve injury, prosthetic complication, hematoma, avascular necrosis, adhesive capsulitis, and instability or dislocation.  ¶: 1 the patient died or had an adverse event during the period 61-365 days following the index PHF, 0 otherwise.  ††: 1 if patient survives 61-365 days after index proximal humerus fracture with less than $300 of shoulder-related healthcare costs, 0 otherwise.  ‡‡: 1 if patient was fully dual-eligible for Medicare and Medicaid during the month of the index PHF, 0 otherwise.  §§: Differences across groups were assessed by the 2-sample independent t test for continuous variables and Pearson χ2 test for categorical data. | | | | |

Table A.1 contains baseline characteristics and benefit and detriment percentages by early treatment choice. As in the prior study, PHF patients receiving early surgery tended to be younger, less frail, and had fewer comorbidities than conservatively managed patients. Early surgery patients were more likely have a detriment outcome and less likely to have a benefit outcome than conservatively managed patients.

| Table A.2: Characteristics of the Study Population by Local Area Early Surgery Ratios | | | | | | | |
| --- | --- | --- | --- | --- | --- | --- | --- |
|  |  | Quintiles of Local Area Early Surgery Ratios (ASRs) | | | | |  |
| Factors | Study Population  (N = 72,751) | 1^st^  (%)  (N=14,908) | 2^nd^  (%)  (N=14,212) | 3^rd^  (%)  (N=14,565) | 4^th^  (%)  (N=15,424) | 5^th^  (%)  (N=13,642) | P value§§ |
| Early Surgery (%) | 16.4 | 10.6 | 14.4 | 16.2 | 18.6 | 22.5 | < 0.001 |
| **Patient-Specific Factors** |  |  |  |  |  |  |  |
| Male | 19.2 | 19.7 | 19.3 | 19.0 | 18.9 | 19.0 | 0.082 |
| Age (66-69) | 13.4 | 12.4 | 13.9 | 13.6 | 13.8 | 13.4 | 0.034 |
| Age (70-75) | 21.1 | 19.8 | 21.0 | 21.4 | 21.2 | 22.1 | < 0.001 |
| Age (76-79) | 15.2 | 15.3 | 15.2 | 15.1 | 15.2 | 15.4 | 0.79 |
| Age (80-85) | 24.9 | 25.6 | 24.2 | 25.2 | 24.6 | 24.7 | 0.17 |
| Age (86 plus) | 25.4 | 26.8 | 25.7 | 24.7 | 25.3 | 24.5 | < 0.001 |
| Asian | 0.9 | 0.8 | 0.6 | 1.2 | 1.5 | 0.3 | 0.52 |
| Black | 3.1 | 2.6 | 3.4 | 2.9 | 3.4 | 3.3 | 0.003 |
| Hispanic | 1.4 | 1.0 | 2.1 | 1.0 | 1.8 | 0.9 | 0.24 |
| Other Race | 1.2 | 1.1 | 1.3 | 1.5 | 1.5 | 0.8 | 0.11 |
| White | 93.4 | 94.5 | 92.6 | 93.4 | 91.8 | 94.7 | 0.27 |
| Fully Dual Eligible‡‡ | 13.5 | 14.9 | 13.3 | 13.1 | 14.8 | 11.0 | < 0.001 |
| CCI (0) | 25.2 | 24.8 | 25.8 | 24.8 | 26.0 | 24.4 | 0.7 |
| CCI (1) | 20.7 | 20.6 | 20.6 | 21.0 | 20.9 | 20.3 | 0.76 |
| CCI (2) | 15.6 | 15.3 | 15.7 | 16.2 | 15.1 | 15.9 | 0.57 |
| CCI (3) | 12.3 | 12.5 | 12.2 | 12.2 | 12.0 | 12.8 | 0.74 |
| CCI (4 plus) | 26.2 | 26.8 | 25.7 | 25.7 | 26.0 | 26.7 | 0.95 |
| FRI (0) | 35.3 | 34.9 | 36.1 | 35.4 | 35.5 | 34.5 | 0.27 |
| FRI (1) | 26.3 | 26.7 | 26.1 | 26.2 | 25.7 | 26.7 | 0.67 |
| FRI (2) | 15.7 | 16.0 | 15.4 | 15.2 | 15.8 | 16.2 | 0.46 |
| FRI (3 plus) | 22.7 | 22.4 | 22.3 | 23.3 | 23.1 | 22.6 | 0.29 |
| Medicare Spending Above Mean Prior to Index† | 26.5 | 27.1 | 25.7 | 26.6 | 26.3 | 26.7 | 0.83 |
| Osteoarthritis | 25.2 | 23.9 | 24.3 | 25.8 | 25.9 | 26.4 | < 0.001 |
| Rheumatoid arthritis | 7.9 | 8.4 | 7.9 | 8.0 | 7.6 | 7.8 | 0.41 |
| Rotator cuff arthropathy | 6.6 | 6.7 | 6.5 | 6.2 | 6.5 | 7.2 | 0.2 |
| Avascular necrosis | 0.2 | 0.2 | 0.2 | 0.2 | 0.2 | 0.2 | 0.71 |
| **County-Level Factors** |  |  |  |  |  |  |  |
| Mean Medicare spending (county) | 9520 | 9448 | 9197 | 9619 | 9659 | 9676 | < 0.001 |
| Mean life expectancy (county) | 78.3 | 78.9 | 78.6 | 77.8 | 78.5 | 77.7 | < 0.001 |
| **Outcomes** |  |  |  |  |  |  |  |
| Death (%)‡ | 10.2 | 9.8 | 10.4 | 10.0 | 10.4 | 10.5 | 0.053 |
| Complication (%)§ | 52.1 | 52.1 | 51.1 | 51.5 | 52.0 | 53.6 | 0.005 |
| Detriment (%)¶ | 56.8 | 56.5 | 56.1 | 56.1 | 57.3 | 58.4 | < 0.001 |
| Benefit (%)† | 58.3 | 56.2 | 57.6 | 59.4 | 58.7 | 59.7 | < 0.001 |
| CCI: Charlson Comorbidity Index score based on Medicare claims in the year prior to index proximal humerus fracture.  FRI: Frailty Risk Index score based on Medicare claims in the year prior to index proximal humerus fracture.  †: 1 if patient had Medicare spending in the 365 days prior to the index PHF that was greater than the average of patients in the sample, 0 otherwise.  ‡: 1 if patient died during the period 61-365 days following the index PHF, 0 otherwise.  §: 1 if patient had an adverse event during the period 61-365 days following the index PHF, 0 otherwise. Adverse events include pneumonia, cardiac dysrhythmias, congestive heart failure, deep vein thrombosis or pulmonary embolism, infection, nerve injury, prosthetic complication, hematoma, avascular necrosis, adhesive capsulitis, and instability or dislocation.  ¶: 1 the patient died or had an adverse event during the period 61-365 days following the index PHF, 0 otherwise.  ††: 1 if patient survives 61-365 days after index proximal humerus fracture with less than $300 of shoulder-related healthcare costs, 0 otherwise.  ‡‡: 1 if patient was fully dual-eligible for Medicare and Medicaid during the month of the index PHF, 0 otherwise.  §§: Trends across early surgery ratio quintiles. Trends in categorical variables were assessed by Cochrane-Armitage test. Trends in continuous variables were assessed by the “t” statistic on an ordinal specifying the quintile in a linear model of the specified variable. | | | | | | | |

Table A.2 distributes patients across quintiles of local area early surgery ratios (ASRs). As in the early study, early surgery percentages across the population range from 10.6% in the first quintile to 22.5% in the fifth quintile. Baseline patient factors such as the CCI, the FRI score, and age, were similar across ASR quintiles. Some statistically significant trends in baseline patient factors remained across quintiles, but for the most part the absolute differences across quintiles were small relative to the comparisons between the early surgery and conservatively managed patients in Table A.1.
